# An Incidental Finding of Unilateral Winged Scapula in a Patient Presenting With Acute Abdomen: A Case Report

**DOI:** 10.7759/cureus.60075

**Published:** 2024-05-11

**Authors:** Aditya Sharma, Parul Khanna, Ruma Kumari

**Affiliations:** 1 Department of General Surgery, Institute of Medical Sciences, Banaras Hindu University, Varanasi, IND; 2 Department of General Medicine, Sri Guru Ram Das University of Health Sciences, Amritsar, IND; 3 Department of Pediatrics, All India Institute of Medical Sciences, New Delhi, IND

**Keywords:** report of a rare case, incidental finding, long thoracic nerve, traumatic injuries, winged scapula

## Abstract

The primary cause of scapular winging, also known as scapula alata, is typically a malfunction of the serratus anterior, trapezius, and rhomboids, the three major scapular stabilizers. Scapular winging is often caused by injuries to the long thoracic nerve, which weakens the serratus anterior muscle. The long thoracic nerve is particularly vulnerable to both acute and nontraumatic damage due to its longer and superficial course. There are very few documented cases of isolated scapula winging. Here, we present the case of a 15-year-old Asian female who initially presented with right hypochondrium pain, and during a general physical examination, an incidental finding of a left-winged scapula was noted.

## Introduction

Scapular winging develops when the serratus anterior, trapezius, and rhomboid major and minor muscles that stabilize the scapula become paralyzed due to various reasons [1]. The most common neurological cause is weakness in the serratus anterior muscles as a result of long thoracic nerve palsy. Long thoracic nerve palsy can have either an atraumatic or traumatic origin [2].

Many other sports have been linked to long-term thoracic nerve palsy in the past. The long thoracic nerve is particularly vulnerable to both acute and nontraumatic damage due to its long and superficial route [3]. After the posterior triangle of the neck surgery, damage to the spinal accessory nerve and consequent trapezius palsy can be seen. A less common reason for scapular winging is the rhomboids not functioning properly [4]. 

## Case presentation

A 15-year-old Asian female initially presented with chief complaints of pain in the abdomen. There was mild tenderness noted in the right hypochondrium. There was a history of an accident where she fell from her bicycle eight months ago. Her past medical history was not significant. She had never previously undergone surgery or been admitted to the hospital. There was no known congenital condition, and her family history was insignificant. Her other systemic examination was within normal limits.

**Figure 1:**
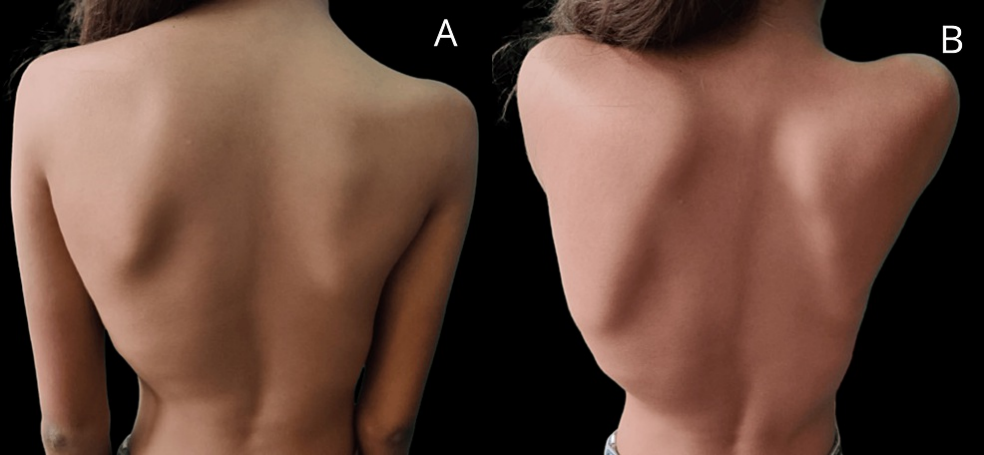
Figure showing an isolated left-winged scapula (A) at rest and (B) during the wall push test.

On general physical examination, an isolated winging of the left scapula was noted. There were no complaints of pain or tenderness during the local examination. The examination findings revealed that there were 60° of external rotation, 90° of active abduction and elevation, and the ability to rotate internally up to D12. The scapulothoracic joint was the primary site of active abduction and elevation. A cervical spine examination revealed no abnormalities. The rotator cuff demonstrated normal strength. Scapular winging was evident both at rest and during the wall push test, as shown in Figure 1 (A and B). 

The pain in the right hypochondrium was relieved and subsided with conservative therapy. An orthopedic consultation was taken for expert opinion. No treatment was suggested by the Department of Orthopedics for the patient's left shoulder because she did not report any symptoms pertaining to the left-winged scapula, and she was kept on periodic follow-up in case any symptoms arise in the future.

## Discussion

Traction injuries have been reported in several cases, as well as direct traumatic injuries to the nerve. Of the injuries to the long thoracic nerve, 26% are due to blunt trauma to the chest wall or the shoulder girdle. Sports like weightlifting, volleyball, archery, and even ballet can cause nerve-stretching problems [3,4]. Scapular winging is a rare side effect of repetitive industrial shoulder use [5]. Scapular winging can be caused by nontraumatic factors such as vaccinations, viral and nonviral diseases, systemic lupus erythematosus, and brachial plexus neuritis (Parsonage-Turner syndrome). However, in certain instances, the cause is unclear [5,6].

We described a case of isolated left scapula winging attributed to a traumatic incident. In 75% of cases, the ventral branch of the fifth cervical spinal nerve in the posterior cervical triangle, deep into the prevertebral fascia, is where the dorsal scapular nerve originates. The nerve shares a trunk with the long thoracic nerve and can get contributions from C4 to T1 on occasion [7]. 

The rhomboid major and minor muscles, as well as occasionally the levator scapulae, are innervated by the dorsal scapular nerve, which pierces the middle scalene muscle and proceeds posteriorly between it and the levator scapulae. Although uncommon, isolated dorsal scapular nerve injuries have been documented [6,8].

## Conclusions

The present case report is rare and hence important as there are very limited case studies reporting the isolated winging of the scapula. Also, this case highlights the importance of general physical examination as in the present case the patient presented with chief complaints of the abdominal system and such important findings were incidentally detected, which could have been missed otherwise. 
